# P-2013. Surgical Site Infections (SSI) are a significant healthcare concern, particularly in patients undergoing clean Lower Segment Caesarean Section (LSCS) surgeries. SSIs can lead to severe complications, extended hospital stays, and increased healthcare costs

**DOI:** 10.1093/ofid/ofaf695.2177

**Published:** 2026-01-11

**Authors:** Dr Arya S Kumar, Nimi Mohan, Geeshma Baby, Dr Sanjeev Singh

**Affiliations:** Department of Infection control & Epidemiology, kochi, Kerala, India; Department of Infection control & Epidemiology, kochi, Kerala, India; Department of Infection control & Epidemiology, kochi, Kerala, India; Department of Infection control & Epidemiology, kochi, Kerala, India

## Abstract

**Background:**

Surgical Site Infections (SSI) are a significant healthcare concern, particularly in patients undergoing clean Lower Segment Caesarean Section (LSCS) surgeries. SSIs can lead to severe complications, extended hospital stays, and increased healthcare costs.

**Figure:**
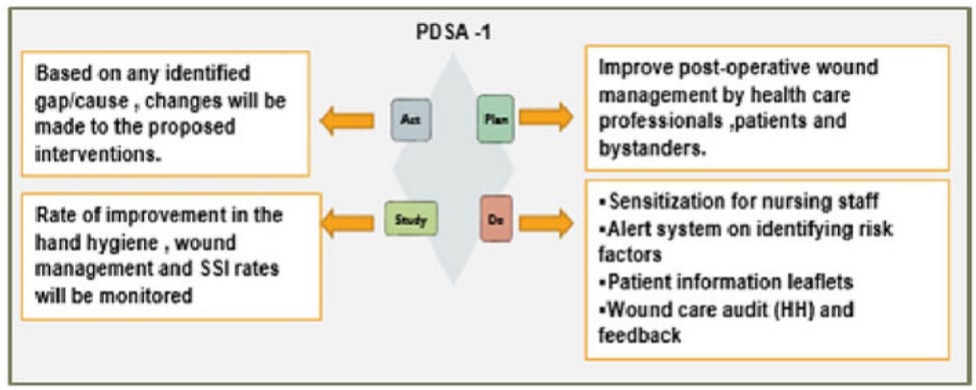

**Figure:**
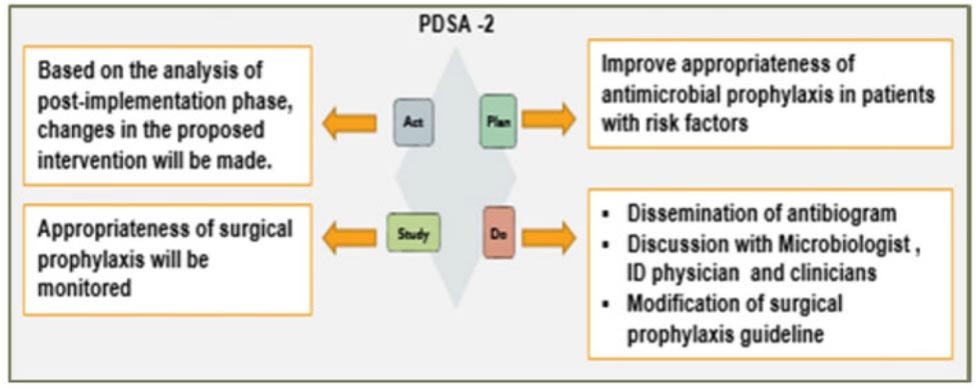

**Methods:**

A multidisciplinary quality improvement team utilized the PDSA model (figure 1& 2) to design and implement strategies aimed at reducing SSIs in LSCS surgeries. They designed two quantitative interventions comprising a care bundle for healthcare professionals, bystanders, and patients, as well as interventions for appropriate surgical prophylaxis and antibiotic regimens for patients with risk factors. The key components of this care bundle include sensitization sessions for nursing staff, an alert system for identifying risk factors, patient leaflets, and wound care audits focusing on hand hygiene.

**Figure:**
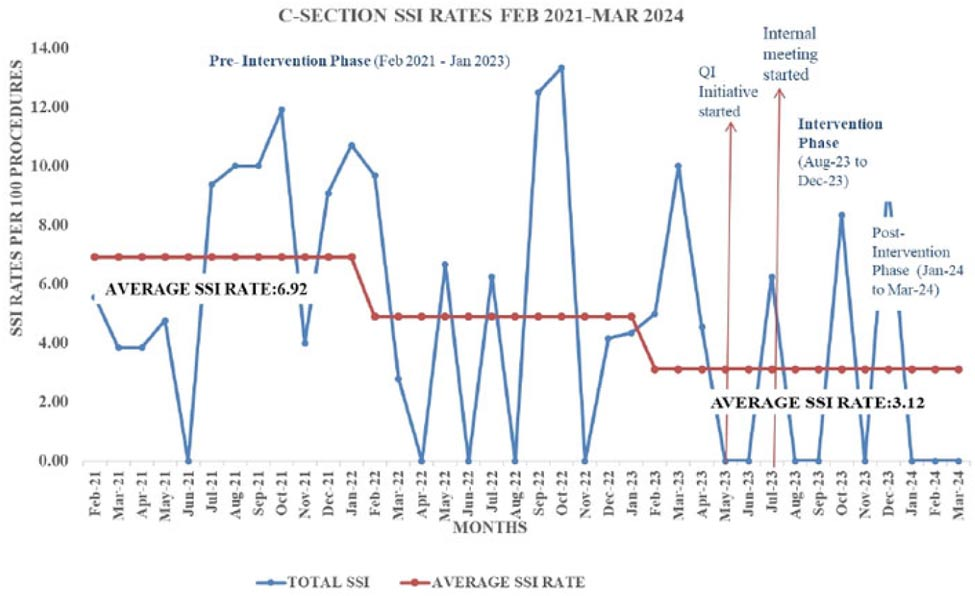
C -SECTION SSI rates

**Results:**

The implementation of care bundle and targeted interventions led to significant reduction in the rate of SSI following caesarean surgery. The rate decreased from 6.69%( from February 2021 to January 2023) to 3.12% within 12 months(figure 3).

**Conclusion:**

This initiative underscores the preventability of SSIs through evidence-based interventions. Key to success were the comprehensive training and sensitization of healthcare staffs, patient and bystander education, and the implementation of standardized prophylactic measures. Sustained quality improvement efforts are vital for maintaining these gains and further enhancing patient outcomes in a tertiary care setting.

**Disclosures:**

All Authors: No reported disclosures

